# In-hospital mortality and major complications related to radiofrequency catheter ablations of over 10 000 supraventricular arrhythmias from 2005 to 2020: individualized case analysis of multicentric administrative data

**DOI:** 10.1093/europace/euac146

**Published:** 2022-08-25

**Authors:** Florian Doldi, Nele Geßler, Omar Anwar, Ann-Kathrin Kahle, Katharina Scherschel, Benjamin Rath, Julia Köbe, Philipp Sebastian Lange, Gerrit Frommeyer, Andreas Metzner, Christian Meyer, Stephan Willems, Karl-Heinz Kuck, Lars Eckardt

**Affiliations:** Department for Cardiology II: Electrophysiology, University Hospital Münster, Albert-Schweitzer-Campus 1 Gebäude A1, D-48149 Münster, Germany; Department of Cardiology and Intensive Care, Asklepios Clinic St. Georg, Hamburg, Germany; Department of Cardiology and Intensive Care, Asklepios Clinic St. Georg, Hamburg, Germany; Klinik für Kardiologie, Angiologie, Intensivmedizin, cNEP Research Consortium EVK, Düsseldorf, Germany; Klinik für Kardiologie, Angiologie, Intensivmedizin, cNEP Research Consortium EVK, Düsseldorf, Germany; Department for Cardiology II: Electrophysiology, University Hospital Münster, Albert-Schweitzer-Campus 1 Gebäude A1, D-48149 Münster, Germany; Department for Cardiology II: Electrophysiology, University Hospital Münster, Albert-Schweitzer-Campus 1 Gebäude A1, D-48149 Münster, Germany; Department for Cardiology II: Electrophysiology, University Hospital Münster, Albert-Schweitzer-Campus 1 Gebäude A1, D-48149 Münster, Germany; Department for Cardiology II: Electrophysiology, University Hospital Münster, Albert-Schweitzer-Campus 1 Gebäude A1, D-48149 Münster, Germany; Universitäres Herz- und Gefäßzentrum UKE Hamburg, Klinik und Poliklinik für Kardiologie, Hamburg, Germany; Klinik für Kardiologie, Angiologie, Intensivmedizin, cNEP Research Consortium EVK, Düsseldorf, Germany; Department of Cardiology and Intensive Care, Asklepios Clinic St. Georg, Hamburg, Germany; Department of Cardiology and Intensive Care, Asklepios Clinic St. Georg, Hamburg, Germany; Department for Cardiology II: Electrophysiology, University Hospital Münster, Albert-Schweitzer-Campus 1 Gebäude A1, D-48149 Münster, Germany

**Keywords:** Interventional electrophysiology, Ablation, Complications, Supraventricular tachycardias, Arrhythmia, Multicentric analysis

## Abstract

**Aims:**

The incidence of in-hospital post-interventional complications and mortality after ablation of supraventricular tachycardia (SVT) vary among the type of procedure and most likely the experience of the centre. As ablation therapy of SVT is progressively being established as first-line therapy, further assessment of post-procedural complication rates is crucial for health care quality.

**Methods and results:**

We aimed at determining the incidence of in-hospital mortality and bleeding complications from SVT ablations in German high-volume electrophysiological centres between 2005 and 2020. All cases were registered by the German Diagnosis Related Groups—and the German Operation and Procedure Classification (OPS) system. A uniform search for SVT ablations from 2005 to 2020 with the same OPS codes defining the type of ablation/arrhythmia as well as the presence of a vascular complication, cardiac tamponade, and/or in-hospital death was performed. An overall of 47 610 ablations with 10 037 SVT ablations were registered from 2005 to 2020 among three high-volume centres. An overall complication rate of 0.5% (*n* = 38) was found [median age, 64; ±15 years; female *n* = 26 (68%)]. All-cause mortality was 0.02% (*n* = 2) and both patients had major prior co-morbidities precipitating a lethal outcome irrespective of the ablation procedure. Vascular complications occurred in 10 patients (0.1%), and cardiac tamponade was detected in 26 cases (0.3%).

**Conclusion:**

The present case-based analysis shows an overall low incidence of in-hospital complications after SVT ablation highlighting the overall very good safety profile of SVT ablations in high-volume centres. Further prospective analysis is still warranted to guarantee continuous quality control and optimal patient care.

**See the editorial comment for this article ‘Catheter ablation of supraventricular tachycardias—a success story’, by Till F. Althoff and Lluís Mont, on pages 4–5.**

**What’s new?**

- Supraventricular tachycardia (SVT) ablations are shown to be a safe treatment option in experienced large volume centres with an overall low incidence of major in-hospital complications including mortality.
- Individual case inspection is a crucial step when analyzing administrative data to avoid misleading assumptions regarding a possible causal relationship between a coded complication and ablation procedure.

## Introduction

Ablation therapy has established itself as first-line therapy for paroxysmal supraventricular tachycardia (SVT). It is therefore crucial to gain further understanding of procedure-related major risks.^1,2^ The incidence of these post-interventional complications and in-hospital mortality varies extensively among the type of procedure and most likely the volume of the centre performing it. Whereas patients that undergo ablation of often complex ventricular arrhythmias in the setting of relevant co-morbidities show relatively high rates of post-interventional complications,^1,3–11^ ablation of SVT is generally regarded as a safe procedure with only rare significant complications. A comprehensive overview of the safety of catheter ablations was published in 2017 by Hosseini *et al*.^2^ analyzing patients in the USA registered by the Nationwide Inpatient Sample database stating an overall complication rate of 5.5%. Although, this study only included patients registered as inpatients and therefore might have some selective bias undercounting simpler procedures, it delivered nationwide insights into the safety of various catheter ablations. Steinbeck *et al*.^11^ analyzed insurance data of patients undergoing atrial fibrillation/atrial flutter ablations in Germany in 2014 and revealed an overall higher rate of major complications than reported in previous studies. To adequately weigh risk and benefit, relative value of the therapy, and evaluate health care quality, a frequent assessment of complication rates of catheter ablations in large unselected nationwide patient cohorts is of major importance.

However, as many reports rely on self-reporting with no information on the individual patient, we aimed at systematically analyzing the incidence and certain predictors of risk for major post-interventional complications and in-hospital mortality on an individual level from catheter ablation procedures for SVT at three German high-volume healthcare centres from 2005 until 2020.

## Methods

**Figure 1:**
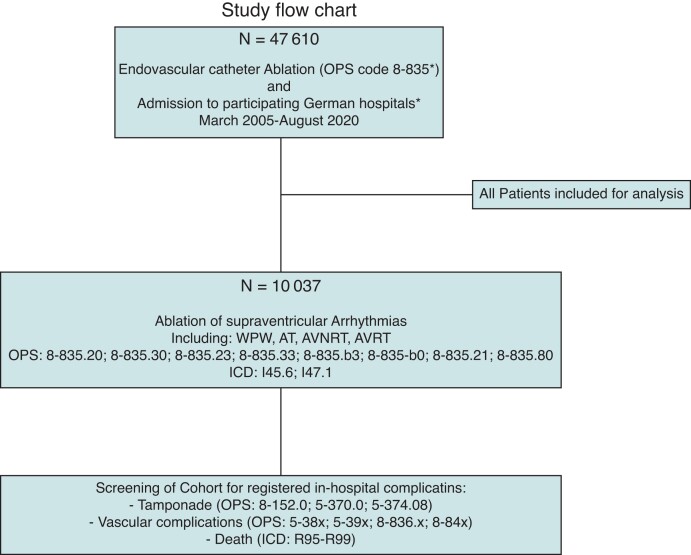
Study flow-chart including inclusion criteria used for analysis. We extracted all patients receiving catheter ablations in participating centres from 2005 to 2020 (*n* = 47 610) and further differentiated the cohort using the mentioned ICD/OPS codes with regard to the type of arrhythmia treated extracting all patients that received catheter-based treatment of a supraventricular arrhythmia (*n* = 10 037). To extract patients that suffered from an in-hospital complication after catheter ablation (*n* = 38), we added OPS codes for specific major complications as mentioned below; (*) serves as a place-holder for further OPS code specifications as mentioned below as well as the participating 3 german high volume centres..

Since 2004, diagnoses and procedures must be transferred to the German Diagnosis Related Groups system (G-DRG). Diagnoses are coded according to the International Statistical Classification of Diseases and Related Health Problems (ICD)-10-GM (German modification), whereas procedures are coded according to the German Operation and Procedure Classification (OPS). As this coding delivers highly differentiated information about the type and location of the targeted cardiac structure, identifying related complications until the day of hospital discharge is possible. In the present retrospective, multicentric nationwide cohort study, all patients who underwent catheter ablation of SVT were registered by the G-DRG system. As DRG-codes do not always comprehensively reflect the type of SVT ablation but merely the location and energy form used, we summarized the cases to a uniform category of ablation of SVT. To ensure uniform results, the three centres prompted a search for all catheter ablation from 2005 until 2020 with the same OPS codes defining the type and location of ablation and arrhythmias. All SVT such as atrioventricular nodal reentry tachycardia (AVNRT), Wolf-Parkinson-White Syndrome/atrioventricular reentry tachycardia, atrial tachycardia (AT) were analyzed. To assess the rate of in-hospital complications after ablation, we added OPS codes describing the presence of an acute bleeding needing surgical vascular intervention, the occurrence of a cardiac tamponade, and death. Thereafter, all patients that suffered from one of the aforementioned in-hospital complications were extracted and individually analyzed case by case for detailed analysis of the relation between the electrophysiologic procedure and the documented complication and to extract clinical data revealing the reason and other circumstances of the documented severe clinical events (*Figure 1*). The study was approved by the institutional ethics review committee. Gathered data were anonymized and consequently analyzed descriptively using R-Studio Version 1.4.1106 (R. RStudio, PBC, Boston, MA, USA).

## Results

The participating three high-volume electrophysiological centres documented 47 610 catheter ablations from 2005 to 2020, of those 10 037 were catheter ablations of SVT. Because of a logistic problem of one clinic’s archives, one centre was only able to extract patient data from 2010 until 2020. Overall, data analysis revealed 38 (0.5%) cases with the combination of SVT ablation and a severe complication such as cardiac tamponade, relevant vascular complication, or death within the same hospital stay (*Table 1*). A median of four venous sheaths (Interquartile Range (QR): 4.0) were placed mostly divided on both femoral veins in 88.9% of the cases (*n* = 24). If an effective anticoagulation was taken at the time of admission it was paused before the procedure in 15.4% of the cases with a relevant bleeding complication (*n* = 2). Median International Normalized Ratio (INR) at the time of the procedure was 1.4 (±0.5). Antagonization of intraprocedural heparin with protamine sulfate (median 5000 Internation Units (IE); SD ± 7250 IE) was deemed necessary in six cases (16%) with a median previous heparin dose of 5000 IE (±2915 IE).

**Table 1:** Overall baseline characteristics of patients with an in-hospital complication after catheter ablation of a supraventricular arrhythmia from 2005 to 2020

Baseline	
*n* (%)	38 (0.5)
Total number	10 037
Age (median SD)	62 (±14)
Sex (f/m)	29/22 (56/43)
BMI	26 (±4)
*Co-morbidities*	
ICM	6 (12)
NICM	5 (10)
LVEF (%)	57 (±8)
ICD (%)	2 (4)
Art. hypertension	31 (60)
DM	6 (12)
Stroke	2 (4)
CHA2DS2Vasc	3 (±2)
OAC (%)	27 (53)
*Mapping/ablation*	
RA	26 (68)
LA	12 (32)
TSP	12 (32)

BMI, body mass index; DM, diabetes mellitus; NICM, non-ischaemic cardiomyopathy; ICM, ischaemic cardiomyopathy; OAC, oral anticoagulation; RA, right atrium; LA, left atrium.

Overall baseline characteristics, procedural, and complicational data are illustrated in *Table 2*.

**Table 2:** In-hospital complications after ablation of a supraventricular arrhythmia from 2005 to 2020

Baseline		Mortality	Vascular interventions	Tamponade
	*N*	2 (0.02)	10 (0.1)	26 (0.26)
	Total number	10 037	10 037	10 037
	Age (median SD)	66 (±2)	67 (±12)	61 (±15)
	Sex (f/m)	−/2 (50)	7/3 (70/30)	14/12 (54/46)
	BMI	25 (±4)	23 (±3)	26 (±4)
*Co-morbidities*				
	ICM	1 (50)	2 (20)	3 (12)
	NICM	1 (50)	2 (20)	0
	LVEF (%)	43 (±25)	55 (±7)	58 (±6)
	ICD (%)	0	1 (10)	0
	Art. hypertension	1 (50)	7 (70)	16 (62)
	DM	2 (100)	0	2 (8)
	Stroke	0	1 (10)	1 (4)
	CHA_2_DS_2_Vasc	3	3 (±1)	2 (±1)
	OAC (%)	0	5 (50)	14 (54)
*Mapping/ablation*				
Location of ablation	RA	2 (100)	5 (50)	19 (72)
	LA	0	5 (50)	7 (27)
	RV	0	—	—
	LV	0	—	—
Ablation method	Epicardial	0	—	—
	TSP	0	5 (50)	7 (27)
	Re-ablation	—	5 (50)	11 (42)
	Radiofrequency	—	4 (40)	26 (100)
Type of arrhythmia	AVNRT	2 (100)	5 (50)	9 (35)
	WPW/AVRT	—	—	3 (12)
	AT	—	5 (50)	14 (54)
*Causes of death*				
Cardiovascular	Cardiogenic shock	2 (100)	—	—
	Stroke	0	—	—
	Tamponade	0	—	—
	Electrical storm	0	—	—
Infectious	Sepsis/pneumonia	0	—	—
Other	Malignancy	0	—	—
*Circumstances of complication*				
Timing	Intraprocedural	0	1 (10)	9 (35)
	Post-procedural	2 (100)	9 (90)	17 (65)
Timing in relation to procedure	Day of procedure	—	2 (20)	24 (92)
	Day after procedure	—	3 (30)	—
	>2 days after procedure	2 (100)	5 (50)	2 (8)

BMI, Body mass index; DM, diabetes mellitus; NICM, non-ischaemic cardiomyopathy; ICM, ischaemic cardiomyopathy; LVEF, left ventricular ejection fraction; ICD , implantable cardioverter defibrillator; OAC, oral anticoagulation; RA, right atrium; LA, left atrium; RV, right ventricle; LV, left ventricle; TSP, transseptal puncture; AVNRT, AV-node reentry tachycardia; WPW, Wolff-Parkinson-White Syndrome; AVRT, atrioventricular reentry tachycardia; AT, atrial tachycardia.

### Supraventricular tachycardia ablation and mortality

Overall, two patients who underwent catheter ablation for SVT therapy died during the hospital stay. A causal relation of both events to the ablation procedures is highly unlikely as both patients were severely diseased before the ablation and the time of death was long after the ablation procedure (Patient 1: 31 days; Patient 2: 11 days). Baseline data and case descriptions of both cases are depicted in *Table 3*.

**Table 3:** Baseline characteristics of patients with a lethal outcome after catheter ablation of a supraventricular arrhythmia

	Age	Sex	BMI	LVEF	Length of stay	Co-morbidities	Type of ablation	Indication	Cause of death
1	67	Male	—	25	40	DM, NICM	Slow-pathway modulation	AVNRT	Septic shock due to severe vascular-related ulcerations
2	64	Male	—	60	11	Hypertension, DM, NICM	Slow-pathway modulation	AVNRT	Cardiogenic shock due to severe pre-existing co-morbidities

DM, diabetes mellitus; NICM, non-ischaemic cardiomyopathy; ICM, ischaemic cardiomyopathy; DCM, dilatative cardiomyopathy; AVNRT, AV-node reentry tachycardia.

*Patient 1*: Sixty-seven-year-old male with a non-ischaemic cardiomyopathy and severely but reduced left ventricular ejection fraction (LVEF 25%) was admitted due to dyspnoea. In addition, he had a high-grade peripheral arterial occlusive disease of both legs (Stage IV) with critical ischaemia and ulcerations and advanced kidney insufficiency on the base of a diabetic nephropathy (chronic kidney disease G4). He was in acute cardiac decompensation with clinical and radiographic signs of fluid. In the presence of major ulcerations of the lower extremities with Gram positive bacteria, a sepsis was diagnosed and treated through antibiotics. Besides, a recanalization attempt of the chronically occluded femoral arteries was unsuccessful. An almost incessant haemodynamically relevant AVNRT worsened the clinical situation so that a successful slow-pathway modulation was performed with no acute complications. Long (>30 days) after the ablation procedure—the patient developed sepsis-related multi-organ failure and died in consequence of his septic shock, despite escalated antibiotic regimens as well as amputation of one extremity.

*Patient 2*: Sixty-four-year-old male with cardiac amyloidosis and severe aortic stenosis presented with dyspnoea and signs of respiratory and cardiac insufficiency associated with an angioedema due to ramipril intake. A symptomatic AVNRT was incidentally diagnosed. The patient underwent slow-pathway modulation after respiratory recompensation. Eleven days after the ablation the patient developed electromechanical decoupling and death most probably due to the underlying cardiomyopathy. There was no evidence for a relation between death and the performed slow-pathway modulation.

### Vascular interventions post-ablation

Ten patients (0.1%) suffered a vascular complication that required surgical intervention. Median age of these patients was 65 ± 15 years with 70% females (*n* = 7) and a body mass index (BMI) of 27 ± 4 kg/m^2^. Only four patients had a cardiomyopathy [non-ischaemic cardiomyopathy, *n* = 2; ischaemic cardiomyopathy (ICM), *n* = 2] and the overall LVEF was 57 ± 4%. One patient had an implanted cardioverter defibrillator, seven patients had arterial hypertension, and one had had a stroke several years ago. Five patients were on oral anticoagulants.

Of these 10 patients, five underwent left atrial ablation. Besides, five patients already had a previous ablation attempt performed for the same arrhythmia. Regarding the timing of these vascular complications, most complications occurred post-procedural (*n* = 9), whereas one significant groin bleeding occurred already during the ablation procedure. For the rest, one bleeding occurred on the day of the procedure and the rest the day after (*n* = 3, 30%) or over 2 days (*n* = 5, 50%) after the procedure. Half of all patients (*n* = 5) were under effective oral anticoagulation at the time of the procedure.

### Cardiac tamponade

Twenty-six patients (0.26%) developed a pericardial tamponade after catheter ablation for SVT. These patients had a median age of 59 ± 13 years, a BMI of 28 ± 7 kg/m^2^ and were mostly female (*n* = 14, 54%) Three patients (12%) had an ICM, 16 (62%) arterial hypertension, 2 (8%) diabetes mellitus, and 1 (4%) a stroke in their medical histories. Fourteen patients (54%) were on oral anticoagulants. Of these 26 patients, 19 catheter ablations (AVNRT *n* = 9; AT *n* = 10) were performed in the right atrium (72%) and seven in the left atrium (LA) (27%). In seven patients (27%), a transseptal puncture was performed. Eleven patients had already been ablated and required re-ablation.

Intraprocedural complications were observed in nine patients (35%) with the remaining 19 occurring post-procedurally. Twenty-four tamponades occurred on the day of the procedure (92%), whereas the remaining two (8%) occurred 2 days after the procedure.

## Discussion

Catheter ablation of patients with paroxysmal SVT is regarded as first-line treatment in current guidelines.^12,13^ Nonetheless, risk for procedural complications related to the invasive nature persist and should be continuously evaluated for quality control and to achieve optimal patient care. Here, we present procedural data on major complications of catheter ablations of SVT in three high-volume centres with each more than 1000 catheter ablations/year over a period of 15 years. Using a systematic automated analysis, a complication rate of 0.5% for severe vascular complications, tamponade, or death was observed. This is at the lower end of the complication rate of 0.3–2.5% among different types of SVT ablations in the literature^1,8,14–16^ including data from the German Ablation registry.^8,17–19^

In accordance with registry data from Singapore (0.6%),^20^ Sweden (0,07%),^15^ Germany (0.03%),^21^ as well as a meta-analysis by Spector *et al*.^22^ (0.1%) and reports by Eugene *et al*. in a 1-year follow-up (0–0.1%); the mortality rate after SVT ablations was low in our long-term study of 15 years SVT ablation with two in-hospital deaths (0.02%). Besides, the individual analysis of these two cases revealed no direct relation of the ablation with the death but severe prior co-morbidities that most probably lead to their demise. Thus, we thereby demonstrate the importance of individual case inspection when analyzing administrative data and possible misleading assumptions being made with regard to a causal association of an in-hospital complication with an underlying ablation procedure. Upon individual case inspection, we can therefore conclude that SVT ablation procedures are safe in the evaluated experienced high-volume centres. Nevertheless, prospective clinical data would of course be of importance for optimal quality control.

As previously shown^23^ vascular complications are the most common adverse events following catheter ablations with a reported incidence of 0.2–1.5% over the years with a slightly higher incidence reported after the ablation of ventricular tachycardias (3.6–6.9%).^20,21,23^ Data on the incidence of vascular complications after electrophysiologic procedures depend on the definition of the outcome (surgical intervention, haematoma, etc.). Sharma *et al*.^23^ for example compared the outcome of patients that received an electrophysiologic procedure with vascular access done by using ultrasound or no ultrasound. All possible major vascular bleeding complications at least meeting the Bleeding Academic Research Consortium (BARC) 2 criteria with and without the need for surgical intervention were included. Here, an overall complication rate of 3.2% has been described with only one patient with an SVT suffering from a vascular complication following the procedure in the ultrasound group.^23^

In our study, we aimed at focusing on major vascular complications that required surgical intervention and could be detected by obligatory administrative data.

Thereby, we identified 10 patients with a vascular complication needing surgical intervention, resulting in a vascular complication rate of 0.1% in the studied three centres. Half of these patients had undergone slow-pathway modulation to treat their AVNRT and another half a catheter ablation of a focal AT. Hence, our data support the generally acknowledged safety of these ablation procedure in comparison with other interventional procedures, e.g. catheter ablations of ventricular tachycardias.

Regarding possible contributing factors to bleeding complications, we analyzed all cases with a vascular complications or cardiac tamponade individually. Twenty-one patients (58%) were on oral anticoagulation with nine patients taking phenprocoumon and 12 non-Vitamin K dependent oral anticoagulants (two apixaban, three dabigatran, five rivaroxaban, two edoxaban) at the time of catheter ablation. Intraprocedurally, these patients did not reveal any distinctive differences with regard to the procedural approach, with most patients receiving ∼5000 IE heparin (±2915 IE) intraprocedurally with protamine sulfate only deemed necessary in six cases (16%) and a normal periprocedural INR (1.4; ±0.5). If an anticoagulation was already indicated and prescribed before the procedure, it was usually continued the day of the procedure in 88.9% of the cases throughout all centres. Of course, the timing of the last intake of oral anticoagulation is of great importance for the incidence of a post-interventional bleeding complication. A meta-analysis by Bawazeer *et al*.^24^ compared the efficacy and harms of interrupted vs. uninterrupted anticoagulation therapy for catheter ablations in adults with arrhythmias and showed uncertain evidence to interrupt or continue the anticoagulation regimen periprocedurally. Therefore, further prospective clinical trials with a patient cohort adopting a more homogenous anticoagulation regimen would be of great interest.

Highlighting this data further, 26 patients (0.26%) of our study developed an intervention-related tamponade after catheter ablation of a SVT. This overall low occurrence is mostly since the majority of SVT (49%) was ablated in the right atrium, therefore, drastically reducing the risk for perforation of the myocardium via a transseptal approach or left atrial mapping. Of note, an overall of nine patients (35%) developed post-procedural tamponade more than 2 h after ablation, again concurring with results from aforementioned prior studies.^22,25,26^ All patients that suffered from haemodynamically relevant pericardial tamponade were transeptally punctured for LA access again highlighting the risk of this procedure and the continuously needed careful evaluation of patients before the procedure.

## Conclusions

Over a 15-year evaluation period ablation of SVT has been shown to be a very safe treatment option in experienced large volume centres. In our retrospective multicentric analysis, we found an overall low incidence of major post-procedural complications after SVT ablations including mortality. Also, we want to highlight the importance of individual case inspection when analyzing administrative data, because of possibly misleading assumptions being made with regard to a causal association a complication with the underlying ablation procedure coded within this hospital stay. Further prospective analysis is still warranted to guarantee optimal quality control and patient care.

### Limitations

The present analysis is based on data from three high-volume EP centres. Therefore, data cannot be generalized. Besides, when analyzing ablation cases, an inherent bias of patient selection in retrospective data collection must be presented. Furthermore, as already stated by prior studies, analysis of administrative data is subject to a high risk of miscoding of ICD codes. However, as every case reported as in-hospital complication was inspected individually by several seasoned physicians to exclude miscoding and relation to procedure. Also, as it is considered common administrative practice in Germany, 15–20% of each coded case are reviewed and evaluated by health insurance agencies to guarantee correct coding and reimbursement. Furthermore, only in-hospital complications were considered for analysis. Therefore, any major life-threatening complication after discharge from the hospital is overlooked.

## Data Availability

The data underlying this article will be shared on reasonable request to the corresponding author.
